# Knockdown of *anterior gradient 2* expression extenuates tumor-associated phenotypes of SNU-478 ampulla of Vater cancer cells

**DOI:** 10.1186/1471-2407-14-804

**Published:** 2014-11-03

**Authors:** Su Jin Kim, Suyeon Jun, Hee-Yeon Cho, Dong Chul Lee, Young Il Yeom, Jong Hyeok Kim, Dongchul Kang

**Affiliations:** Departments of Internal Medicine, Hallym University Sacred Heart Hospital, Hallym University College of Medicine, Anyang, Korea; Department of Internal Medicine, Sejong General Hospital, Bucheon, Korea; Department of Biomed Gerontol and Ilsong Institute of Life Science, Hallym University, Anyang, Korea; Korea Research Institute of Bioscience and Biotechnology, Daejeon, Korea

**Keywords:** AGR2, Tumor promotion, SNU-478, SNU-869, Biliary tract cancer, Ampulla of Vater

## Abstract

**Background:**

Anterior gradient 2 (AGR2) has been implicated in tumor-associated phenotypes such as cell viability, invasion and metastasis in various human cancers. However, the tumor promoting activity of AGR2 has not yet been determined in biliary tract cancers. Thus, we examined the expression of AGR2 and its tumor-promoting activity in biliary tract cancer cells in this study.

**Methods:**

Expression of AGR2 mRNA and protein was analyzed by real time RT-PCR and western blotting, respectively. MTT assay was employed to measure cell viability and pulsed BrdU incorporation by proliferating cells was monitored by flow cytometry. Soft agar colony formation assay and transwell invasion assay were employed to determine anchorage-independent growth and *in vitro* invasion of the tumor cells, respectively. *In vivo* tumor formation was examined by injection of tumor cells into immunocompromised mice subcutaneously. Statistical analysis was performed with 2-tailed unpaired Student’s *t*-test for continuous data and with one-way ANOVA for multiple group comparisons. Bonferroni tests were used for *post hoc* 2-sample comparisons.

**Results:**

*AGR2* mRNA was detected in SNU-245, SNU-478, and SNU-1196 cell lines, and its protein expression was confirmed in SNU-478 and SNU-245 cell lines by western blot analysis. Knockdown of *AGR2* expression with an *AGR2*-specific short hairpin RNA (shRNA) in SNU-478, an ampulla of Vater cancer cell line resulted in decreased cell viability and in decreased anchorage-independent growth by 98%. The AGR2 knockdown also increased the sensitivity of the cells to chemotherapeutic drugs, including gemcitabine, 5-fluorouracil and cisplatin. In addition, SNU-478 cells expressing *AGR2*-shRNA failed to form detectable tumor xenografts in nude mice, whereas control cells formed tumors with an average size of 179 ± 84 mm^3^ in 3 weeks. Overexpression of AGR2 in SNU-869 cells significantly increased cell viability through enhanced cell proliferation and the number of Matrigel™-invading cells compared with AGR2-negative SNU-869 cells.

**Conclusions:**

Our findings implicate that AGR2 expression augments tumor-associated phenotypes by increasing proliferative and invasive capacities of the ampulla of Vater cancer cells.

## Background

*Anterior gradient 2* (*AGR2*, also known as *hAG-2*) is a human orthologue of the *Xenopus laevis* cement gland-specific gene *XAG-2* that functions in specifying dorsoanterior ectodermal fate, including formation of cement glands and induction of forebrain fate in *Xenopus* [1–3]. Human and murine AGR2 (*Gob-4)* expression was first identified in estrogen receptor (ER)-positive breast cancer cells and in goblet cells of the stomach, small intestine and colon, respectively [2, 3]. AGR2 that belongs to the protein disulfide isomerase (PDI) family containing an atypical thioredoxin fold (CXXS) is essential for the production of MUC2 protein in the intestine [4].

Specific expression of AGR2 in ER-positive breast cancer cells suggests that AGR2 plays a role in the pathogenesis of ER-positive cancers [3]. Although correlation of AGR2 expression with ER is further supported by estrogen-dependent induction of AGR2 expression [5], AGR2 expression is not restricted to ER-positive cancer cells. AGR2 has been found to be expressed highly in diverse human cancers, including adenocarcinomas of the esophagus [6], lung [7], pancreas [8], ovary [9] and prostate [10]. AGR2 promotes metastasis of breast cancer, hepatocellular carcinoma and head and neck squamous carcinoma cells [11–13]. Presence of AGR2 protein in serum and AGR2 expression in circulating tumor cells have been reported in patients of ovarian and lung cancer [9, 14]. Moreover, AGR2 expression is implicated in tamoxifen resistance of breast cancer, probably due to tamoxifen-induced AGR2 expression [15].

Tumor promoting role of AGR2 *in vitro* and *in vivo* has been demonstrated in various contexts. Overexpression of AGR2 augments many important features of cancer cells including proliferation, survival, metastasis and drug resistance (reviewed in detail by [16, 17]). Conversely, knocking down of AGR2 expression decreases cell growth and induces cell death in ER-positive breast cancer cells [18]. Silencing of AGR2 expression in MPanc-96 pancreatic cancer cell line significantly decreases tumor growth in a xenogeneic tumor model [19]. Moreover, AGR2-expressing NIH3T3 cells produce tumors in nude mice [20]. These results clearly manifest the functionality of AGR2 in tumorigenesis and tumor progression.

Cancers of the biliary tract are anatomically heterogeneous diseases arising at the bile duct (intrahepatic and extrahepatic cholangiocarcinomas including Klatskin tumor), gall bladder and ampulla of Vater [21]. Because of the nonspecific symptoms of the disease and aggressive nature of the tumor, biliary tract cancers are often diagnosed at advanced stages and, thus highly lethal. Although incidence, gender bias and cure rates vary depending on primary tumor sites, overall 5-year survival rate is 5 ~ 10%, and 25 ~ 30% with curative surgery [22]. Despite that AGR2 is implicated in tumorigenesis and tumor progression of various cancers, AGR2 expression and its tumor-promoting role in biliary tract cancers have not yet been studied in detail. AGR2 is reported to be expressed in normal tissues of the biliary tract and the expression pattern is conserved in biliary tract cancer [23]. However, the expression and tumor-promoting function of AGR2 in biliary tract cancer cells have not been investigated to date. Thus, this study aimed to analyze the expression and functional role of AGR2 in development and maintenance of tumor phenotypes of biliary tract cancer cells. To this end, we determined AGR2 expression in six biliary tract cancer cell lines. In addition, tumor-promoting activity of AGR2 was examined by knockdown of AGR2 expression with shRNA and its overexpression in AGR2-positive SNU-478 and AGR2-negative SNU-869 ampulla of Vater cancer cell lines, respectively.

## Methods

### Biliary tract cancer cell lines

Six human biliary tract cancer cell lines (SNU-245, SNU-308, SNU-478, SNU-869, SNU-1079 and SNU-1196) and MCF-7 breast cancer cells were procured from the Korea Cell Line Bank (Seoul, Korea) [24]. The seven carcinoma cell lines were cultured in RPMI-1640 medium supplemented with 10% fetal bovine serum, 100 U/mL penicillin, and 100 μg/mL streptomycin in a humidified incubator at 37°C in an atmosphere of 5% CO_2_. The cell lines were subcultured by splitting at 1:8 ratios twice weekly.

### Cell viability and BrdU incorporation assays

Cell viability and drug sensitivity were examined by the MTT assay. The biliary tract cancer cells were plated in a 96-well plate at 4000 cells/well for SNU-869 or 2000 cells/well for SNU-478 to compensate for the different growth rates of the individual cell lines. Both SNU-478 and SNU-869 cells were plated at 8000 cells/well in a 96-well plate for drug sensitivity test. MTT solution (0.5 mg/mL, Sigma-Aldrich, St. Louis, MO, USA) was added to each well at 72 h after plating for growth analysis and after drug treatment for drug sensitivity test, and MTT formazan was dissolved with lysis buffer as described by Huynh et al. [25]. MTT conversion was measured by absorbance at 570 nm with a reference absorbance at 650 nm using a microplate spectrophotometer (Multiskan GO, Thermo Scientific, Rockford, IL, USA).

Cell proliferation was analyzed by BrdU incorporation assay as described in Verma et al. [26] with minor modification. SNU-869 stable transfectants were plated at 3 × 10^5^ cells in a 10-cm culture plates. The cells were grown for four days and pulsed with 20 μM BrdU for 30 min. Harvested cells by trypsinization were washed with PBS containing 1% FBS twice and fixed in 70% ethanol for one hour at -20°C. After washing with PBS, the fixed cells were permeabilized by incubation in 2 M HCl for one hour at room temperature and then washed with PBS three times. The cells were divided into two aliquots and treated with FITC-α-BrdU (BD Biosciences, San Jose, CA, USA) or FITC-mouse IgG_1_ κ isotype control (eBioscience, San Diego, CA, USA) in dark for 45 min at room temperature. Then, the cells were washed with PBS twice and resuspended in 500 μL of 1.5 μg/mL 7-AAD in PBS. Fluorescence signals of FITC-α-BrdU and 7-AAD were measured by flow cytometry using a FACSCalibur™ (BD Bioscience), and the data were analyzed with CellQuest Pro software (BD Bioscience).

### Quantitative real-time reverse-transcription (RT) PCR

A TRIzol reagent (JBI, Daegu, Korea) was used to extract total RNA from the biliary tract cancer cells grown to ~70% confluence. The total RNA (5 μg) was used in reverse transcription reactions in 20-μL reaction mixtures with ImProm-II™ (Promega, Madison, WI, USA), dNTPs, and an oligo(dT) primer according to the vendor’s protocol. Real-time PCR was carried out with the reverse-transcribed cDNA (2 μL) using the SYBR^®^ Green PCR Kit (Qiagen, Hilden, Germany) in Light Cycler 2.0 (Roche, Basel, Switzerland) as directed by the manufacturer’s protocol. Primers used in the experiments were the followings: AGR2 forward, 5′-ATGGAGAAAATTCCAGTGTC-3′; AGR2 reverse, 5′-TTACAATTCAGTCTTCAGCA-3′; glyceraldehyde-3-phosphate dehydrogenase (GAPDH) forward 5′-TGATGACATCAAGAAGGTGGTGAAG-3′; and GAPDH reverse 5′-TCCTTGGAGGCCATGTGGGCCAT-3′. PCR was performed with the following cycling protocol: 1 cycle of 95°C for 10 min, followed by 45 cycles of 95°C for 10 s, 56°C for 5 s, and 72°C for 20 s. *AGR2* expression was normalized against the expression of *GAPDH*.

### Western blot analysis

Cells grown to 70% ~ 80% confluence were harvested by scraping and lysed in RIPA buffer (50 mM Tris-HCl pH 7.5, 150 mM NaCl, 1% NP-40, 0.5% sodium deoxycholate, 1 mM EDTA, and 0.1% SDS, freshly supplemented with 1 mM DTT and protease-inhibitor cocktails). Protein concentration was measured by the BCA method (Thermo Scientific, Rockford, IL, USA). Protein samples (30 μg) were resolved by 12% SDS-PAGE and electroblotted onto a nitrocellulose membrane. Immunodetection was carried out with αAGR2 antibody (rabbit polyclonal, 1:3000 dilution, Imgenex Corp., San Diego, CA, USA). The same blot was reprobed for β-actin to monitor protein loading on the blot.

### Establishment of stable transfectants

pLKO.1-puro-based short hairpin RNA (shRNA) expression vectors targeting *AGR2* expression or a vector control (Sigma-Aldrich, St. Louis, MO, USA) was transfected into SNU-478 cells using Lipofectamine^®^ 2000 (Invitrogen, Carlsbad, CA, USA). Transfected SNU-478 cells with the shRNA control, shAGR2-1, shAGR2-2, or shAGR2-3 were selected by treatment with puromycin (2.5 μg/mL, Sigma-Aldrich, St. Louis, MO, USA) to establish stable *AGR2*-knockdown cells. To generate stable AGR2-overexpressing and control transfectants, the AGR2-expression vector (pcDNA3.1-AGR2) was transfected into SNU-869 cells, using Lipofectamine^®^ 2000. AGR2 stable transfectant clones of SNU-869 were selected by treatment with 0.4 mg/mL G418 (Invitrogen, Carlsbad, CA, USA). Knockdown or overexpression of AGR2 in the stable transfectants was verified by RT-PCR and western blotting. The SNU-478 AGR2-knockdown cells and SNU-869 AGR2 stable transfectants were maintained in medium containing puromycin (2.5 μg/mL) and G418 (0.4 mg/mL) until further analysis, respectively.

### Soft agar colony-formation assay

SNU-478 cells stably transfected with the vector control or *AGR2* shRNA were harvested by trypsinization, and 1000 cells were mixed in 0.3% top agar and plated onto 0.6% base agar in 6-cm culture dishes. Cells in the agar were fed twice weekly and maintained for 3 weeks. Colonies visible in the top agar were counted directly.

### Transwell invasion assay

The transwell invasion assay was performed with Biocoat™ Matrigel™ Invasion Chambers with 8-μm pores (BD Biosciences) as directed by the vendor’s protocol. AGR2-knockdown SNU-478 or AGR2-overexpressing SNU-869 cells (5 × 10^4^ cells/well) were resuspended in 200 μL serum-free RPMI medium and then added to the upper chamber. Bottom chambers were filled with 1% fetal bovine serum-RPMI, and the cells were incubated for 24 h at 37°C in a 5% CO_2_ incubator. Cells present in the coated membrane were fixed with 3.7% formaldehyde in PBS, permeabilized with 100% methanol for 20 min, and stained with 1% crystal violet for 1 h. The membrane was detached, wiped with a cotton swab, and examined under the microscope at 100 × magnification (Olympus, Tokyo, Japan).

### Formation of tumor xenografts in athymic nude mice

*AGR2*-knockdown SNU-478 cells and control transfectants were harvested by trypsinization, washed twice in PBS, and resuspended to a final concentration of 1.0 × 10^7^ cells/mL. Cell suspensions (0.1 mL) were injected into the subcutaneous tissue of the back of 3 mice (6-wk-old male athymic BALB/c-Slc-*nu/nu*) per test group. The resultant tumors were measured with calipers and the volume was calculated with the formula, length × width × depth/2 [27]. Animals were sacrificed after observation for 21 days and tumor masses were resected. All animal experiments were approved by the Institutional Animal Use and Care Committee of the Korea Research Institute of Bioscience and Biotechnology (KRIBB) and were performed in accordance with the Guide for the Care and Use of Laboratory Animals published by the US National Institutes of Health (NIH Publication No. 85-23, revised 1996).

### Statistical analysis

Statistical analysis of quantitative data was performed with 2-tailed unpaired Student’s *t*-tests (for continuous data) and with the 2-tailed Fisher’s exact test (for categorical data). Multiple group comparisons were assessed by one-way ANOVA. Bonferroni tests were used for *post hoc* 2-sample comparisons.

## Results

### AGR2 is differentially expressed in biliary tract cancer cell lines

**Figure 1:**
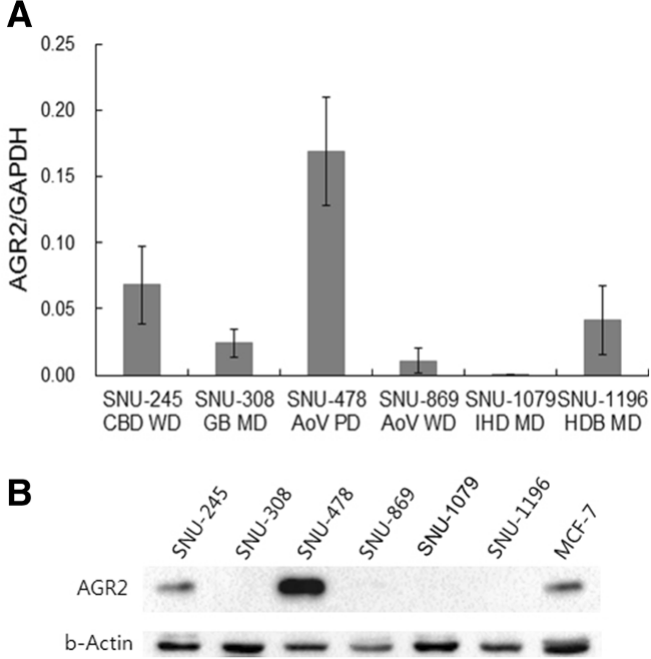
**AGR2 expression in cancer cell lines of the biliary tract. A**. *AGR2* mRNA levels in six biliary tract cancer cell lines were measured by real-time RT-PCR. The *AGR2* transcript levels were normalized against those of the *GAPDH*. Results shown are means ± standard deviations of (1/2)^CT of target - CT of GAPDH^ of three experiments. **B**. AGR2 protein expression was detected by western blot analysis with β-actin as a loading control. The result shown is a representative of three experiments. Abbreviations: CBD, common bile duct; GB, gall bladder; AoV, ampulla of Vater; IHD, intrahepatic duct; HDB, hepatic duct bifurcation; WD, well differentiated; MD, moderately differentiated; PD, poorly differentiated [24].

The expression of AGR2 was examined at the levels of mRNA and protein by real time RT-PCR and western blot analysis, respectively, in the six human biliary tract cancer cell lines that were established from adenocarcinomas of distinct differentiation status and anatomical locations [24]. Relative levels of *AGR2* mRNA decreased in the cell lines in the following order: SNU-478, -245, -1196, -308, -869, and -1079 (Figure 1A). Expression of the AGR2 protein was confirmed in the SNU-478 and -245 cells (Figure 1B). AGR2 expression level in SNU-245 was comparable to that in MCF-7 cells (Figure 1B). The AGR2 expression pattern in the biliary tract cancer cell lines did not appear to correlate with location or differentiation status of the original cancer tissue directly.

### Reversion of tumor phenotypes by knockdown of AGR2 expression

**Figure 2:**
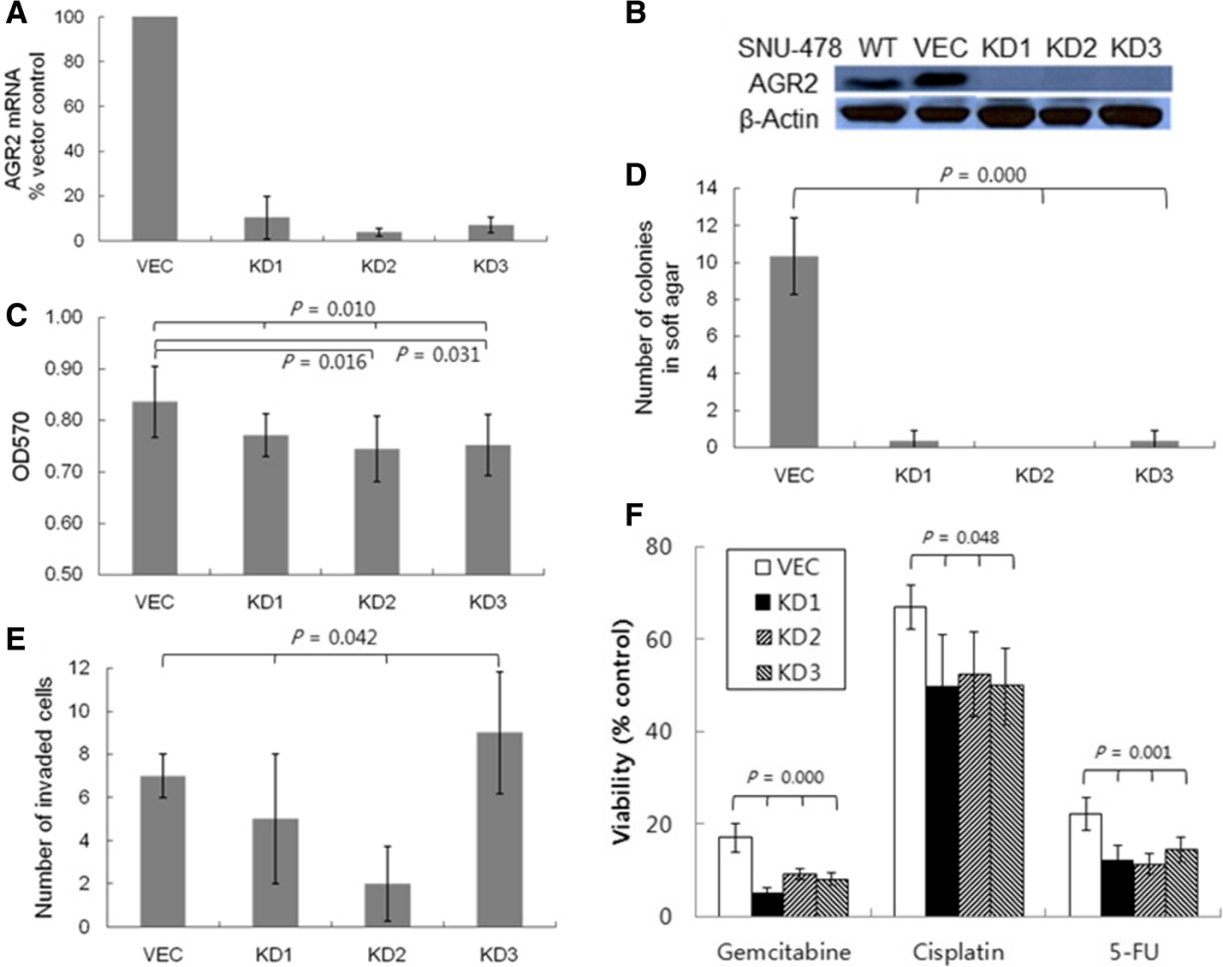
**Alterations of tumor-associated phenotypes in AGR2-silenced SNU-478 cells. A**. Expression of *AGR2* mRNA in SNU-478:KD (*AGR2* knockdown) clones was compared with that in SNU-478:VEC (vector control) by real-time RT-PCR. The *AGR2* transcript level was normalized against that of *GAPDH*. Results shown are means ± standard deviations of (1/2)^CT of target - CT of GAPDH^ of three experiments. **B**. AGR2 protein expression in SNU-478:VEC and SNU-478:KDs was detected by western blot analysis with β-actin as a loading control. The result shown is a representative of three experiments. **C**. Viability of SNU-478:VEC and SNU-478:KDs was measured by the MTT assay. Results shown are means ± standard deviations of three experiments (ANOVA *P* = 0.010; *post hoc* analysis by Bonferroni testing: KD2 vs. VEC, *P* = 0.016). **D**. Anchorage-independent growth was examined by colony formation assay in soft agar. The number of colonies formed in the soft agar assay with SNU-478:VEC and SNU-478:KDs. Results shown are means ± standard deviations of three experiments (ANOVA *P* = 0.000; *post hoc* analysis by Bonferroni testing: KD1-3 vs. VEC, *P* = 0.000). **E**. The number of invading cells in the transwell invasion assay with SNU-478:VEC and SNU-478:KDs. Results shown are means ± standard deviations of three experiments (ANOVA *P* = 0.042). **F**. Drug sensitivity of SNU-478:VEC and SNU-478:KDs was measured by the MTT assay. Cells plated in a 96-well plate were treated with gemcitabine (20 μg/mL), cisplatin (0.5 μg/mL), or 5-FU (50 μg/mL), and the MTT assay was carried out on day three of the treatments. (ANOVA *P* = 0.000 for gemcitabine; ANOVA *P* = 0.048 for cisplatin; ANOVA *P* = 0.001 for 5-FU).

The SNU-478 cell line consists of poorly differentiated cells that were established from an adenocarcinoma at the ampulla of Vater [24]. SNU-478 exhibited the highest level of AGR2 expression and the fastest growth among the six biliary tract cancer cells. Therefore, we used the SNU-478 cells to investigate the tumor promoting role of AGR2 by silencing its expression with an *AGR2* shRNA. Compared with *AGR2* expression in cells transfected with the vector control (SNU-478:VEC), the expression of *AGR2* mRNA was knocked down by 79% ~ 98% in three stable transfectants expressing *AGR2* shRNA (SNU-478:KD1 ~ 3; Figure 2A). AGR2 protein was undetectable in the *AGR2* shRNA-expressing cells by western blot analysis (Figure 2B). The effect of AGR2 expression on cell viability was assessed with the SNU-478:KD cells by the MTT assay. Knockdown of AGR2 expression significantly decreased the viability of the SNU-478:KD2 and KD3 cells, and moderately decreased the viability of the SNU-478:KD1 cells (Figure 2C). Overall, knockdown of AGR2 expression in the SNU-478:KD cells decreased cell viability by 7 ~ 13% compared to the SNU-478:VEC, suggesting an association of AGR2 expression with cell growth and survival.

Colony-forming ability in soft agar was compared among the SNU-478:VEC and SNU-478:KD cells to determine the effect of AGR2 expression on anchorage-independent growth. Knockdown of AGR2 expression dramatically decreased the number of colonies on soft agar. SNU-478:KD cells formed 98% fewer colonies in soft agar than the SNU-478:VEC cells (Figure 2D). AGR2 is known to enhance invasiveness of tumor cells [9, 19, 20]. Therefore, modulation of invasiveness by AGR2 was examined by transwell invasion assays with the SNU-478:VEC and SNU-478:KD cells. Unlike the results of the soft agar colony-forming assay, the AGR2 knockdown resulted in mixed responses among the KD clones and did not significantly inhibit invasion of the tumor cells. However, SNU-478:KD cells tended to be less invasive than SNU-478:VEC cells (Figure 2E), suggesting a weak association of AGR2 expression with tumor invasiveness. On the basis of the above results of the soft agar colony-forming and the transwell invasion assays, AGR2 expression is assumed to augment tumor-specific phenotypes including anchorage-independent growth and invasiveness.

Knockdown of AGR2 expression has been shown to increase the sensitivity of MPanc-96 cells to gemcitabine [19]. Therefore, MTT assay was used to investigate whether knockdown of AGR2 expression altered drug sensitivities of SNU-478 cells (Figure 2F). Knockdown of AGR2 rendered the SNU-478:KD cells significantly more sensitive to gemcitabine, cisplatin and 5-fluorouracil (FU) than the SNU-478:VEC cells. SNU-478:KD cells were ~57% more sensitive to gemcitabine, ~24% to cisplatin, and ~43% to 5-FU than the SNU-478:VEC cells. AGR2 knockdown affected sensitivity of the cells to gemcitabine more severely than those to cisplatin and 5-FU.

**Figure 3:**
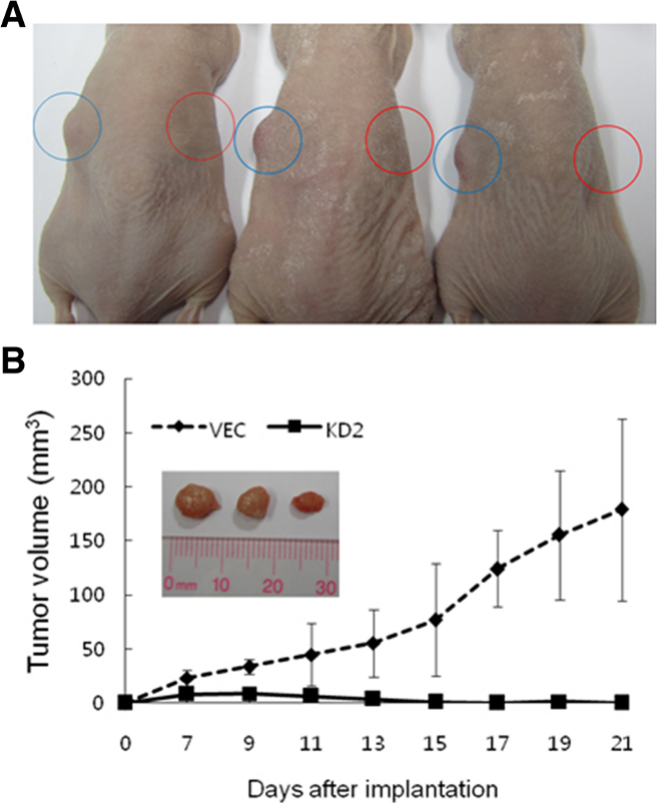
**The effect of the** ***AGR2*** **knockdown on** ***in vivo*** **tumor formation of SNU-478 cells.** *In vivo* tumor formation was examined by subcutaneously injecting SNU-478:VEC or SNU-478:KD2 cells into athymic nude mice. **A**. Mice were injected with SNU-478:VEC (left side) or SNU-478:KD2 (right side) cells and nursed for 21 days. **B**. Increase in tumor size measured after a week post injection as volume (mm^3^) every two days for 21 days. Results shown are means ± standard deviations of three mice (Student’s *t*-test, *P* = 0.021). **B-insert**. Resected tumors formed by SNU-478:VEC after 21 days post injection.

Finally, we examined the effect of AGR2 expression on *in vivo* tumor formation by injecting an equal number of SNU-478:VEC and SNU-478:KD2 cells into BALB/c-Slc-nu/nu immunocompromised mice. SNU-478:KD2 manifested all of the phenotypic characteristics resulted from knockdown of AGR2 expression in SNU-478:KD cells representatively (Figure 2A). Tumor xenografts were formed after injection with the SNU-478:VEC cells, but no palpable tumor mass was detected in mice injected with the SNU-478:KD2 cells (Figure 3A,B insert). The tumor mass of the SNU-478:VEC cells continued to increase until 21 days post injection to 179.00 ± 84.06 mm^3^, whereas the tumor mass of the SNU-478:KD2 cells briefly increased in the first week and, then, regressed over time (Figure 3B). The observed failure of the SNU-478:KD2 cells to form tumor clearly supports a tumor-promoting role of AGR2 expression *in vivo*.

### Augmentation of tumor phenotypes by overexpression of AGR2

**Figure 4:**
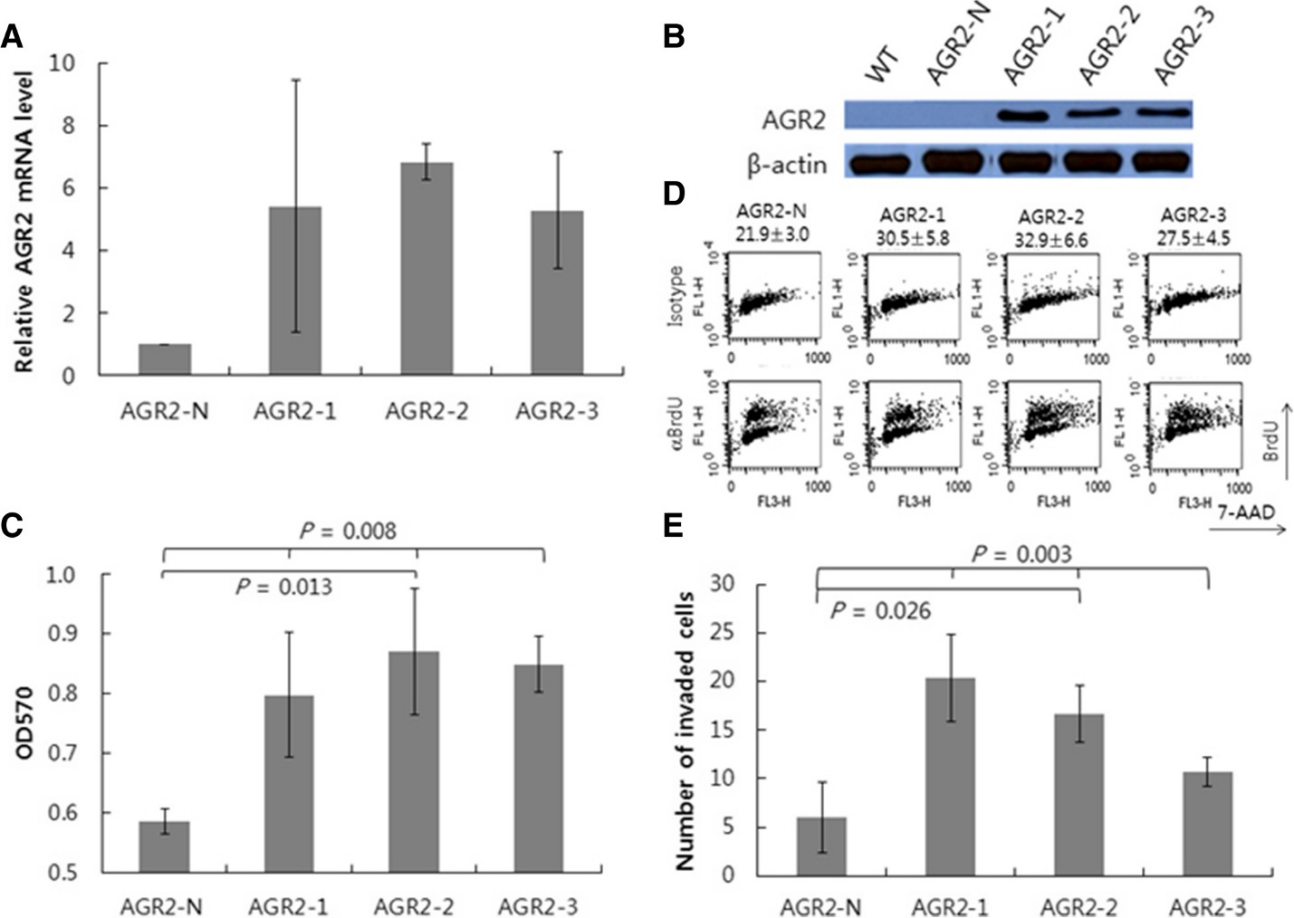
**Alterations of tumor-associated phenotypes by overexpression of AGR2 in SNU-869 cells. A**. *AGR2* expression in the SNU-869 stable transfectants was verified by real-time RT-PCR. The *AGR2* transcript level was normalized against that of *GAPDH*. Results shown are means ± standard deviations of (1/2)^CT of target - CT of GAPDH^ of three experiments. **B**. AGR2 protein expression in the SNU-869 stable transfectants was detected by western blot analysis with β-actin as a loading control. The result shown is a representative of three experiments. **C**. Viability of the SNU-869 stable transfectants was measured by the MTT assay. Results shown are means ± standard deviations of three experiments (ANOVA *P* = 0.008; *post hoc* analysis by Bonferroni testing: AGR2-2 vs. AGR2-N, *P* = 0.013). **D**. The effect of AGR2 overexpression on cell proliferation. Pulsed BrdU incorporation into the SNU-869 stable transfectants was measured by flow cytometry. Percentage of BrdU-positive cells was shown in mean ± standard deviation of four experiments (ANOVA *P* = 0.053; *post hoc* analysis by Bonferroni testing: AGR2-2 vs. AGR2-N, *P* = 0.027). Results shown are representatives of four experiments. **E**. The effect of AGR2 overexpression on tumor cell invasion of SNU-869 cells. The numbers of invading cells in the transwell invasion assay were determined for SNU-869 stable transfectants. Results shown are means ± standard deviations of three experiments (ANOVA *P* = 0.003; *post hoc* analysis by Bonferroni testing: AGR2-2 vs. AGR2-N, *P* = 0.026).

Knockdown of AGR2 expression in SNU-478 cells suppressed certain tumor phenotypes, including growth, anchorage-independent growth, and *in vivo* tumor xenograft formation. To further confirm the AGR2 effects on the development of these tumor phenotypes, AGR2 was overexpressed in the SNU-869 cell line, another ampulla of Vater cancer cell line which shows negligible expression of AGR2. In contrast to SNU-478, SNU-869 is a well-differentiated adenocarcinoma cell line [24]. Three stable AGR2-overexpressing transfectants (SNU-869:AGR2-1 ~ 3) and one AGR2-negative clone (SNU-869:AGR2-N) were established and expression of AGR2 in the transfectants was confirmed at the levels of mRNA and protein by RT-PCR and western blotting, respectively (Figure 4A and B).

The viability of the SNU-869 stable transfectants measured by the MTT assay showed that AGR2 expression significantly increased cell viability (Figure 4C). The viability of the SNU-869:AGR2 clones was on average ~42% higher than that of SNU-869:AGR2-N, providing further support for the growth- and/or survival-enhancing effects of AGR2. In order to determine the mechanism of the elevated cell viability in the SNU-869:AGR2 cells, cell proliferation rate was measured by pulsed BrdU incorporation assay. Percentage of BrdU-positive cells was 8.4% higher on average in 7-AAD-positive SNU-869:AGR2 cells compared to 7-AAD-positive SNU-869:AGR2-N (*P* = 0.053, Figure 4D). Moreover, percent BrdU-positive cells normalized against SSC were 8.8% higher on average in SNU-869:AGR2 cells compared to SNU-869:AGR2-N (21 ± 2.4 in AGR2-N vs. 30.0 ± 5.9, 26.9 ± 4.1 and 32.4 ± 6.3 in AGR2-1 ~ 3, respectively, *P* =0.036). These results suggest that the enhanced proliferation rate could account for the viability increase of the SNU-869:AGR2 cells.The effect of AGR2 on the invasiveness of tumor cells was examined by the transwell migration assay with the SNU-869:AGR2 and SNU-869:AGR2-N cells (Figure 4E). Invasion of the tumor cells was significantly increased by AGR2 overexpression in the SNU-869:AGR2 cells. The number of invading cells was increased on average by about 3-fold in SNU-869:AGR2 over SNU-869:AGR2-N. Thus, AGR2 overexpression seems to enhance the viability and invasiveness of the SNU-869 cells. However, in contrast to the results with the SNU-478:KD cells, overexpression of AGR2 in SNU-869 cells did not result in prominent changes in anchorage-independent growth and drug sensitivities (data not shown).

## Discussion

AGR2 enhances several tumor-specific phenotypes including cell proliferation, survival, anchorage-independent growth, invasiveness through Matrigel™ and metastasis [13, 19, 20]. Drug resistance of cancer cells against gemcitabine and tamoxifen is also affected by AGR2 expression [19, 28, 29]. Knockdown of AGR2 expression in SNU-478 cells lowered the viability of the cells, increased their sensitivity to chemotherapeutic drugs, and decreased anchorage-independent growth significantly. Moreover, AGR2 expression in SNU-869 cells significantly increased cell viability and Matrigel™ invasiveness. AGR2 expression in SNU-869 enhanced cell proliferation rate that should result in the increased cell viability measured by the MTT assay as in normal mammary epithelial cells and in various cancer cells [9, 13, 18, 26]. Thus, AGR2 expression is supposed to augment the tumorigenic potential of the biliary tract cancer cells by increasing the viability, invasiveness and anchorage-independent growth of these cells as was observed in other cancer cells [13, 19].

AGR2 is also positively correlated with *in vivo* tumor-forming ability in athymic nude mice and metastasis of tumor cells. Knockdown of AGR2 expression in MPanc-96 pancreatic cancer cell line and in SEG-1 esophageal adenocarcinoma cell line results in significant reduction of *in vivo* tumor growth [19, 20]. In addition, rat mammary tumor cells overexpressing AGR2 showed increased metastatic potential when injected orthotopically in syngeneic rats [11]. No detectable tumor xenograft was formed in nude mice injected with *AGR2*-silenced SNU-478 cells, whereas *AGR2*-expressing SNU-478 cells formed palpable tumors. Loss of *in vivo* tumorigenic potential of SNU-478 cells after AGR2 knockdown suggests that AGR2 expression is required to establish and to maintain stable tumor xenograft of the biliary tract cancer cells.

However, both knockdown of AGR2 expression in SNU-478 and AGR2 overexpression in SNU-869 were only partially effective in altering certain cancer phenotypes and affected distinct properties in each cell line. Knockdown of AGR2 in SNU-478 cells had only marginal effects in the Matrigel™ invasion assay. In addition, AGR2 expression in SNU-869 cells did not increase anchorage-independent growth, drug resistance and formation of tumor xenograft significantly. Despite that both SNU-478 and SNU-869 cells were originated from tumors of the ampulla of Vater, they were different from each other in their AGR2 expression, differentiation status, growth kinetics and drug responses [24]. Therefore, the phenotypic difference between SNU-478 and SNU-869 might account for the distinct responses to modulation of AGR2 expression in the two cells. Otherwise, partial reversion of the cancer phenotypes by modulation of AGR2 expression could also implicate that AGR2 by itself is insufficient and requires presence of additional cell specific component(s) to cause the necessary change.

The mechanism underlying the AGR2-associated tumor promotion has been being investigated recently. AGR2 silences UV-induced p53 transactivation activity by attenuating p53 phosphorylation in H1299 cells [6]. In addition, overexpression of AGR2 upregulates genes that are involved in cell proliferation, invasion and angiogenesis [9]. Meanwhile, knockdown of AGR2 expression results in downregulation of cyclin D1, survivin, and c-Myc [18]. Moreover, AGR2 expression is suppressed by SMAD4 that is a tumor suppressor of pancreatic ductal adenocarcinoma [30]. In addition, induction of AGR2 expression by tamoxifen through AKT or Src has been implicated in tamoxifen resistance of breast cancer cells [31]. Both AGR2-mediated inhibition of p53 tumor suppressor activity and AGR2-associated expression of genes that regulate cell growth and survival may directly contribute to the tumor-promoting activity of AGR2 through augmented cell proliferation and survival. Meanwhile, increased invasiveness and metastasis associated with AGR2 expression might be the result of regulation of cathepsins B and D expression, enhanced angiogenesis and modulation of extracellular matrix by AGR2 [30, 32, 33]. It is plausible that changes in the AGR2-associated gene expression regulate the tumor phenotypes of the biliary cancer cells including viability, anchorage-independent growth and invasiveness. Therefore, initial clues to determine the mechanism of the AGR2-enhanced tumorigenic potential and drug resistance of the biliary cancer cells can be obtained by detailed investigation in the AGR2-associated alterations in gene expression pattern.

Although AGR2 expression was found to enhance tumor-associated phenotypes of the biliary tract cancer cells, caution should be taken to correlate these results with tumor progression and prognosis of biliary tract cancer patients directly. An immunohistochemical analysis of AGR2 expression pattern demonstrated that AGR2 expression in the biliary tract is not tumor specific, but is associated with anatomical location and mucin-secreting phenotype [23]. An independent study on AGR2 expression in tissue specimens of biliary tract cancer patients revealed that AGR2 expression was higher in tumors of lower stage and advanced differentiation [34]. Such discrepancy between *in vitro* tumor-promoting role of AGR2 and tumor progression in patients has also been reported in pancreatic cancer studies. Whereas knockdown of AGR2 in pancreatic cancer cells decreases cell proliferation and invasion, and increases drug sensitivity [19], AGR2 expression in tissue samples of pancreatic cancer patients is positively correlated with differentiation status of the cancer [35]. In this case, aberrant AGR2 expression in poorly differentiated cancer is correlated with worse prognosis of the patients, suggesting that tumor promoting role of AGR2 is specific to tumor type and stage [35]. Lack of correlation between *in vitro* tumor-promoting activity of AGR2 and pathologic findings in biliary tract cancer patients is an issue to be resolved. It is conceivable that uncoupling of AGR2 expression from tumor progression of biliary tract cancers can be ascribed to peculiarity of biliary tract cancers including cancer microenvironment. Since AGR2 manifested tumor promoting activity in ampulla of Vater cancer cells, pathologic relevance of AGR2 expression in carcinogenesis of the ampulla of Vater should also be investigated in detail. An orthotopic biliary tract cancer model in which AGR2 expression can be modulated specifically in the biliary tract might be required to provide more definitive evidences on *in vivo* tumor-promoting function of AGR2 in biliary tract cancers.

## Conclusions

By analyzing AGR2 expression and its tumor-promoting role *in vitro* and *in vivo*, we have provided evidences for tumor-promoting activities of AGR2 in ampulla of Vater cancer cells for the first time. AGR2 is thought to promote tumor formation by augmenting cell viability, anchorage-independent growth, and invasive properties of the biliary tract cancer cells. The mechanisms underlying the AGR2-associated tumor promotion in the biliary tract cancer cells and the observed discrepancy in the tumor promoting potential of AGR2 in different cells remain to be elucidated in future studies in which AGR2 knockdown SNU-478 or AGR2 overexpressing SNU-869 could be utilized as a valuable model system.
